# Human *SHQ1* variants R335C and A426V lead to severe ribosome biogenesis defects when expressed in yeast

**DOI:** 10.3389/fgene.2023.1240416

**Published:** 2023-09-25

**Authors:** Ismaël Alidou-D’Anjou, Aniket Patel, Sophie Sleiman, François Dragon

**Affiliations:** Centre d’Excellence en Recherche sur les Maladies Orphelines—Fondation Courtois (CERMO-FC), Départment des Sciences Biologiques, Université du Québec à Montréal, Montréal, QC, Canada

**Keywords:** ribosome biogenesis, ribosomal RNA processing, small nucleolar RNPs, SHQ1, dystonia

## Abstract

SHQ1 is an essential chaperone that binds the pseudouridine synthase dyskerin in the cytoplasm and escorts the enzyme to the nucleus, where dyskerin is assembled into small nucleolar RNPs (snoRNPs) of the H/ACA class. These particles carry out pseudouridine formation in ribosomal RNAs (rRNAs) and participate in maturation of rRNA precursors (pre-rRNAs). Variants of human SHQ1 have been linked to neurodevelopmental deficiencies; here we focused on two compound heterozygous mutations identified in a child showing a severe neurological disorder comprising cerebellar degeneration. To investigate the molecular defects caused by mutations R335C and A426V we used a conditional yeast strain that can be depleted of the endogenous Shq1 protein while constitutively expressing human SHQ1 (wild-type or variants). Although wild-type SHQ1 complemented the Shq1-depleted strain, cells expressing variant R335C could not support growth, and cells expressing variant A426V were temperature-sensitive. When shifted to restrictive conditions, yeast cells progressively lost H/ACA snoRNAs and accumulated unprocessed pre-rRNAs, which led to reduced production of ribosomes. Levels of Cbf5 (yeast homologue of dyskerin) were decreased in yeast cells expressing SHQ1 variants under restrictive conditions. Immunoprecipitation experiments revealed that interaction of Cbf5 with SHQ1 variants was weakened but not abolished, and yeast two-hybrid assays showed that mutation R335C is more deleterious than mutation A426V. Our data provide additional evidence for the critical role of SHQ1 in chaperoning the pseudouridine synthase dyskerin, and how its inadequate function has detrimental consequences on the production of H/ACA snoRNPs and ribosomes.

## 1 Introduction

Ribosomes are sophisticated macromolecular machines that synthesize proteins in all living organisms; they are composed of several ribosomal proteins and few ribosomal RNAs (rRNAs), which are the catalytic components of ribosomes (Lafontaine and Tollervey, 2001). Synthesis of eukaryotic rRNAs involves RNA polymerases I and III. In the nucleolus, RNA pol I transcribes a long precursor molecule of 47S (35S in yeast), which is then processed into three smaller rRNAs of 18S, 5.8S and 28S (25S in yeast; Supplementary Figure S1); in the precursor, these three rRNAs are separated by internal transcribed spacers ITS1 and ITS2, the 18S rRNA sequence is preceded by the 5′ external transcribed spacer (5′-ETS) and the 3′-ETS follows the 25/28S rRNA sequence (Panov et al., 2021). RNA pol III transcribes the 5S rRNA precursor in the nucleoplasm (Ciganda and Williams, 2011). Making functional ribosomes requires precise processing of pre-rRNAs as well as coordinated assembly of ribosomal subunits: over 200 non-ribosomal factors are recruited to pre-rRNAs in a hierarchical manner, having roles in RNA processing, modification, folding, RNP assembly, and export of immature subunits to the cytoplasm (Kressler et al., 2010; Woolford and Baserga, 2013; Sharma and Lafontaine, 2015; Bassler and Hurt, 2019; Klinge and Woolford, 2019).

Post-transcriptional modifications in rRNAs are necessary for optimal ribosome function. Notably, several modified residues lie in functional regions of the ribosome (Decatur and Fournier, 2002; Sharma and Lafontaine, 2015). The two major types of rRNA modifications in eukaryotes are 2′-O-methylation and pseudouridylation, the conversion of uridine into pseudouridine (Ψ). These modifications are made by small nucleolar ribonucleoproteins (snoRNPs). C/D snoRNPs add a methyl group at the 2′ position of specific riboses, whereas H/ACA snoRNPs form Ψs (Sloan et al., 2017). Few snoRNPs are involved in processing reactions, such as such as U3, U8 and U14 (C/D class), or U17/snR30 (H/ACA). The U3, U14 and snR30 snoRNPs are essential for 18S rRNA production in yeast (Maxwell and Fournier, 1995; Watkins and Bohnsack, 2012).

Pseudouridylation is catalyzed by a key component of H/ACA snoRNPs, the pseudouridine synthase dyskerin (Garus and Autexier, 2021). In addition to dyskerin (also named DKC1 or NAP57; Cbf5 in yeast), each H/ACA snoRNA associates with three conserved proteins (GAR1, NHP2, and NOP10) following a multi-step assembly process (Massenet et al., 2017). Upon its synthesis in the cytoplasm dyskerin interacts with the chaperone SHQ1, which forms a grip-like structure on the snoRNA binding interface (PUA domain) of the enzyme. This high-affinity interaction protects dyskerin from degradation and non-specific RNA binding (Yu and Meier, 2014). The dyskerin-SHQ1 dimer travels to the nucleus where dyskerin further binds the nuclear assembly factor NAF1 together with NHP2 and NOP10, and SHQ1 is removed from dyskerin with the assistance of AAA + ATPases to allow RNP assembly at the site of snoRNA transcription; the ultimate assembly step is the substitution of NAF1 with GAR1 (Yu and Meier, 2014; Massenet et al., 2017; Schlotter et al., 2023).

**FIGURE 1:**
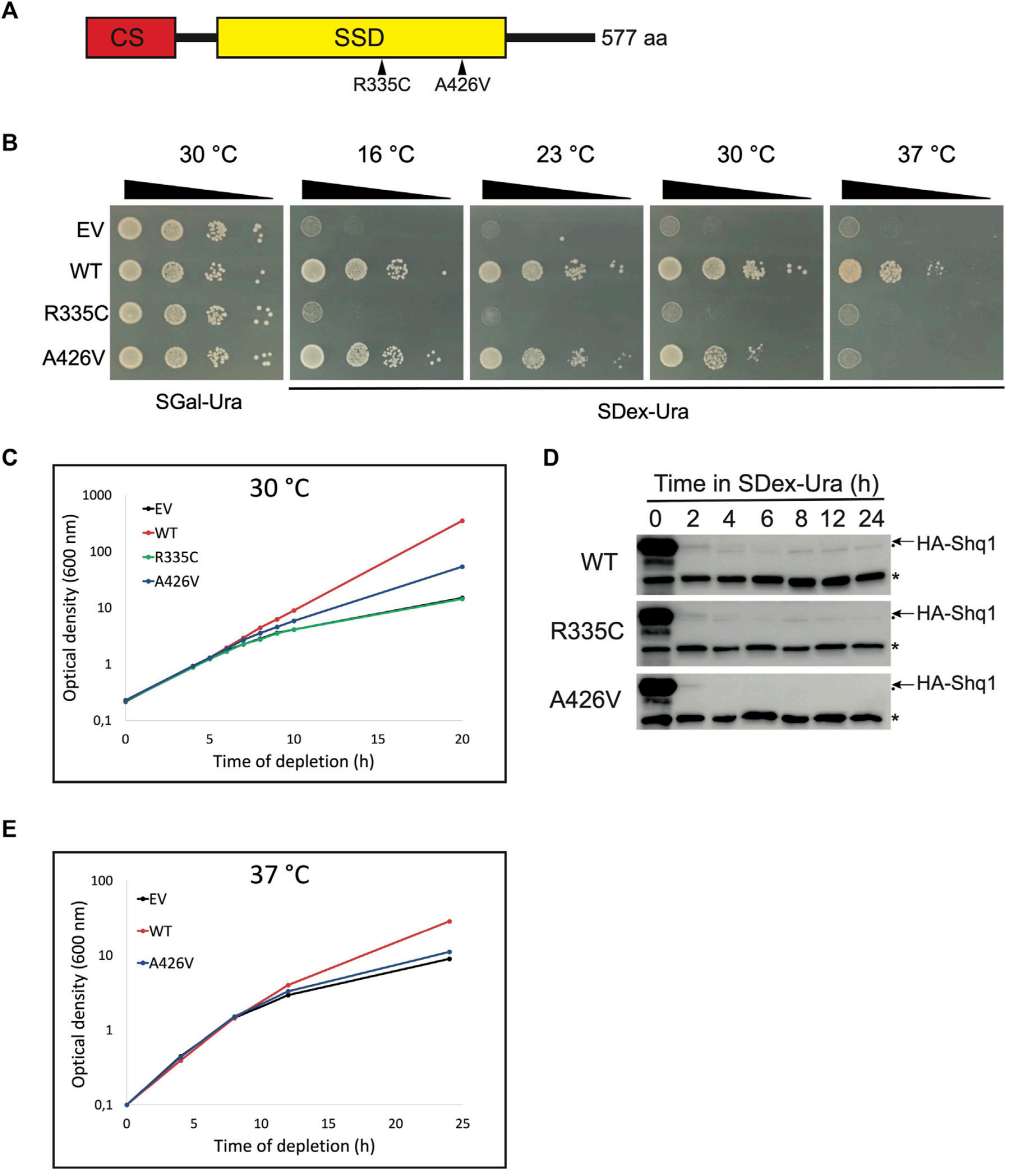
Expression of human SHQ1 variants alters yeast growth. **(A)** Schematic representation of human SHQ1 protein, which comprises two structural domains: a N-terminal CHORD-containing proteins and Sgt1 (CS) domain shown in red, and a SHQ1-specific domain (SSD) in yellow. The position of point mutations in the SSD is indicated by arrowheads. **(B)** Growth assays of conditional yeast strain YSO12 transformed with pCM-FLAG (empty vector; EV) or constructs encoding wild-type hSHQ1 (WT) or its variants (R335C and A426V). Exponentially growing cells were spotted in 10-fold dilutions on a SGal-Ura plate (permissive condition allowing expression of endogenous yeast Shq1) and incubated at 30°C. Cells were spotted similarly on SDex-Ura plates (restrictive condition), and plates were incubated at different temperatures indicated above the plates. **(C)** Growth curves at 30°C. Strains described in **(B)** were cultivated to exponential phase in SGal-Ura at 30°C, shifted to SDex-Ura medium, and growth at 30°C was monitored by measuring the OD600 at various time points. **(D)** Immunodetection of chromosome-encoded endogenous HA-tagged yeast Shq1. Strains described in **(B)** were cultivated in SGal-Ura to exponential phase and shifted to SDex-Ura to deplete HA-Shq1. Aliquots of the cultures were harvested at the indicated times after the shift to SDex-Ura, and whole cell extracts were prepared for western analyses with 12CA5 anti-HA mAb to detect HA-Shq1 (indicated by the arrow). The 12CA5 mAb also recognizes an unknown yeast protein of 50 kDa (indicated by an asterisk), which is used as internal loading control. Another, very faint, non-specific band was also detected and is indicated by a dot below the arrow. **(E)** Growth curves at 37°C. Strains were cultivated in SGal-Ura at 30°C, shifted to prewarmed SDex-Ura, and growth at 37°C was monitored as in **(C)**.

Defects in ribosome assembly or function can lead to a variety of diseases, such as Diamond-Blackfan anemia, Shwachman-Diamond syndrome, dyskeratosis congenita or Treacher Collins syndrome, for example,. Such diseases have been grouped under the term ribosomopathies (Narla and Ebert, 2010; Armistead and Triggs-Raine, 2014; Yelick and Trainor, 2015; Aubert et al., 2018; Farley-Barnes et al., 2019; Venturi and Montanaro, 2020). Compound heterozygous variants in *SHQ1* have recently been linked to early onset dystonia and other neurological features; when expressed in yeast cells, these mutated forms of SHQ1 impaired formation of H/ACA snoRNPs and ribosomes (Sleiman et al., 2022). A previous report also described *SHQ1*compound heterozygous variants in a child with severe neurological disorder including cerebellar degeneration; this individual carried variants c.1003C>T (p.R335C) and c.1277C>T (p.A426V) (Figure 1A), and pulldown assays with recombinant proteins revealed that each mutation reduced binding of SHQ1 to dyskerin (Bizarro and Meier, 2017). To this day, no detailed study was carried out to examine the impact of these mutations at the molecular or cellular levels. Here we used our previously described yeast system (Sleiman et al., 2022) to investigate the effects of variants R335C and A426V *in vivo*. We show that mutations in SHQ1 alter the SHQ1-Cbf5 interaction *in vivo* and lead to altered snoRNA accumulation, pre-rRNA processing, and ribosome formation.

## 2 Materials and methods

### 2.1 Yeast strains and media

*Saccharomyces cerevisiae* strains are listed in Supplementary Table S1. Transformation of yeast cells was made by the lithium acetate/single-stranded carrier DNA/polyethylene glycol method (Gietz and Woods, 2002). Strain YSO12 (Sleiman et al., 2022) was transformed with plasmid constructs to express human SHQ1 and its variants; transformants were maintained in synthetic medium (0.17% yeast nitrogen base, 0.5% ammonium sulfate) containing 2% galactose but lacking uracil (SGal-Ura). To deplete endogenous Shq1 protein, cells were grown to exponential phase at 30°C in SGal-Ura, quickly washed with sterile water, and switched to prewarmed SDex-Ura medium containing 2% dextrose instead of galactose. Yeast two-hybrid assays were done with strain AH109 (Clontech) transformed with constructs made with plasmids pGBKT7 and pGADT7 (Clontech).

### 2.2 Plasmids

Plasmid pCM-hSHQ1-FLAG, a derivative of pCM188 bearing a *URA3* marker (Garí et al., 1997), allows constitutive expression of human SHQ1 with a C-terminal FLAG tag in yeast (Sleiman et al., 2022). Point mutations were introduced in pCM-hSHQ1-FLAG by site-directed mutagenesis (Higuchi et al., 1988) to generate plasmids encoding human SHQ1 variants R335C and A426V (pCM-R335C-FLAG and pCM-A426V-FLAG). Using these plasmids, the coding sequence of human *SHQ1* and its variants was amplified by PCR and subcloned into plasmid pGADT7 (*LEU2* marker) to generate pGAD-hSHQ1, pGAD-R335C and pGAD-A426V. A portion of human *DKC1* gene was amplified by PCR from plasmid pDKC1 (Trahan and Dragon, 2009) and cloned into pGBKT7 (*TRP1* marker) to produce pGBK-miniDKC1∆cat; this plasmid encodes a truncated human dyskerin lacking regions that are not required for interaction with SHQ1, including the N-terminal residues 1 to 46, the catalytic domain comprising residues 151 to 251 (replaced by a single Gly residue), and the C-terminal residues 409 to 514. Sequences of oligonucleotides used for cloning are available on request. All constructs were verified by automated sequencing at the Centre d’expertise et de services Génome Québec.

### 2.3 Complementation assays

Cells carrying different pCM-SHQ1-FLAG constructs were grown to exponential phase in SGal-Ura, washed two times with sterile water and brought to an OD_600_ of 1.0 before making 10-fold serial dilutions in sterile water. Drops of 3 µL were spotted on SGal-Ura and SDex-Ura plates (2% agar), which were incubated at temperatures ranging from 16°C to 37°C until single colonies were visible (around 2–3 days). To monitor growth in liquid medium, strains were cultivated to exponential phase in SGal-Ura at 30°C, washed twice in sterile water, transferred to prewarmed SDex-Ura medium and incubated with shaking at 30°C or 37°C. Growth was monitored by measuring the optical density at 600 nm (OD600) at various time points. To keep cultures in exponential growth, they were repeatedly diluted with pre-warmed SDex-Ura medium and maintained at OD600 < 0,8.

### 2.4 Yeast two-hybrid (Y2H) assays

Strain AH109 was co-transformed with the bait plasmid pGBK-miniDKC1∆cat and a prey plasmid (pGAD-hSHQ1, pGAD-R335C or pGAD-A426V). Transformants were selected on SDex-Trp-Leu plates, and Y2H assays were conducted on SDex-Trp-Leu-His plates supplemented with various concentrations of 3-amino-1,2,4-triazole (3-AT; Sigma). Plates were incubated for 4 days at 30°C.

### 2.5 Immunoprecipitation experiments

Exponentially growing cells in SGal-Ura were shifted to SDex-Ura and cultivated at 30°C for 2 h. The equivalent of 10 A_600_ units of cells were used for each immunoprecipitation (IP) experiment. Whole cell extracts were prepared in TMN100 buffer (25 mM Tris-HCl [pH 7.9], 10 mM MgCl_2_, 100 mM NaCl, 0.1% NP-40, 1 mM 1,4-dithiothreitol [DTT] and supplemented with cOmplete™ protease inhibitor cocktail [Roche]) by mechanical disruption with glass beads, as described (Soltanieh et al., 2015). The extracts were incubated with anti-FLAG M2 affinity gel (Sigma) at 4°C on a nutator for 2 h, then washed 5 times with TMN100 buffer. Beads were resuspended in 50 µL of 2× Laemmli sample buffer and incubated at 95°C for 5 min. Eluted proteins were analyzed by SDS-PAGE and Western blotting.

### 2.6 Immunoblotting

After SDS-PAGE proteins were transferred onto polyvinylidene difluoride membranes (Millipore). Membranes were incubated with primary and secondary antibodies for 1 h with agitation at room temperature. Primary antibodies were mouse anti-myc monoclonal antibody (mAb) from 9E10 hybridoma supernatant, mouse anti-FLAG M2 mAb (Sigma; F1804), and mouse anti-HA mAb from 12CA5 hybridoma supernatant. The secondary antibody was HRP-conjugated anti-mouse IgG (Cytiva; NA931). Membranes were treated with Immobilon Western Chemiluminescence HRP Substrate (Millipore) and signals were visualized with a Fusion FX7 imaging system. Bands were quantified with ImageJ (Schneider et al., 2012).

### 2.7 Sucrose density gradients

Cells were lysed in TMK100 buffer (same as TMN100 buffer, but KCl was substituted for NaCl) as described above for IPs. The extract (7 A_260_ units) was loaded onto 7%–47% linear sucrose density gradients and fractionated with an ISCO density gradient fractionator as described (Lemay et al., 2011) to record polysome profiles.

### 2.8 RNA extraction and Northern blotting

Extraction of total RNA and small RNAs was carried out as described (Soltanieh et al., 2014). Small RNAs were separated by migration in denaturing 8% polyacrylamide gels. For large RNAs, migration was done in 1.2% formaldehyde-agarose gels. RNAs were transferred onto nylon membranes (Cytiva), hybridized with 5′-end labeled probes (Supplementary Table S2) as described (Soltanieh et al., 2015), and blots were exposed to a phosphor screen.

## 3 Results

### 3.1 Mutations in hSHQ1 induce growth defects

To examine the possible molecular defects caused by point mutations R335C and A426V in human SHQ1 (hSHQ1) we used the conditional yeast strain YSO12 that expresses endogenous *SHQ1* from the galactose-inducible *GAL1* promoter: when cells are cultivated in galactose-containing medium they express HA-tagged yeast Shq1 protein but shifting the culture to dextrose-containing medium inhibits production of endogenous Shq1. Because *SHQ1* is an essential gene, survival of Shq1-depleted yeast cells depends on complementation by constitutively expressed, plasmid-borne hSHQ1 (Sleiman et al., 2022). Strain YSO12 was transformed with different pCM-FLAG constructs (Supplementary Table S1) and tested for complementation on selective plates (Figure 1B). All strains grew normally on SGal-Ura medium (permissive condition), but differences appeared on SDex-Ura plates: as expected, the strain carrying the empty vector (EV) did not grow, but the strain expressing wild-type hSHQ1 (WT) grew at all temperature tested. In contrast, no complementation with variant R335C was observed, suggesting that this mutation was lethal. The strain expressing variant A426V grew at low temperatures but not at 37°C, revealing the temperature-sensitivity of this strain. We also examined the behavior of the various strains when cultivated in liquid medium at 30°C (Figure 1C). Growth curves corroborated the phenotypes seen on agar plates: the WT strain grew normally, whereas growth rate of the EV strain began to decrease 6 h after the shift to SDex-Ura medium and reduced even more at later time points. The behavior of strain R335C was identical to EV, further indicating that mutation R335C was lethal. As seen on agar plates incubated at 30°C, strain A426V showed reduced growth rate compared to WT. In parallel, we monitored the levels of endogenous HA-tagged yeast Shq1 during incubation in SDex-Ura (Figure 1D). At time 0 h, the signal for HA-Shq1 was intense in all strains due to over-expression form the very strong *GAL1* promoter, but it reduced rapidly once the strains were shifted to dextrose-containing medium. Indeed, levels of HA-Shq1 largely decreased within 2 h after the shift to SDex-Ura, but the growth rate of the mutants was not affected at this time point (Figure 1C). It took around 6 h of depletion to observe a slowing of growth (Figure 1C), a time point where HA-Shq1 was barely detectable (Figure 1D). Thus, the low levels of HA-Shq1 detected at early depletion times were sufficient to properly chaperone Cbf5 and maintain normal growth, and reduction of growth rate occurred once levels of HA-Shq1 were inadequate. We next examined the behavior of strain A426V cultivated in liquid medium at 37°C (Figure 1E): the growth curve of this strain was analogous to strain EV, indicating that mutation A426V was lethal at 37°C. Note that the WT strain had reduced growth at 37°C, consistent with previous observations on agar plates (Sleiman et al., 2022).

### 3.2 Weakened interaction of SHQ1 variants reduces the levels of Cbf5

**FIGURE 2:**
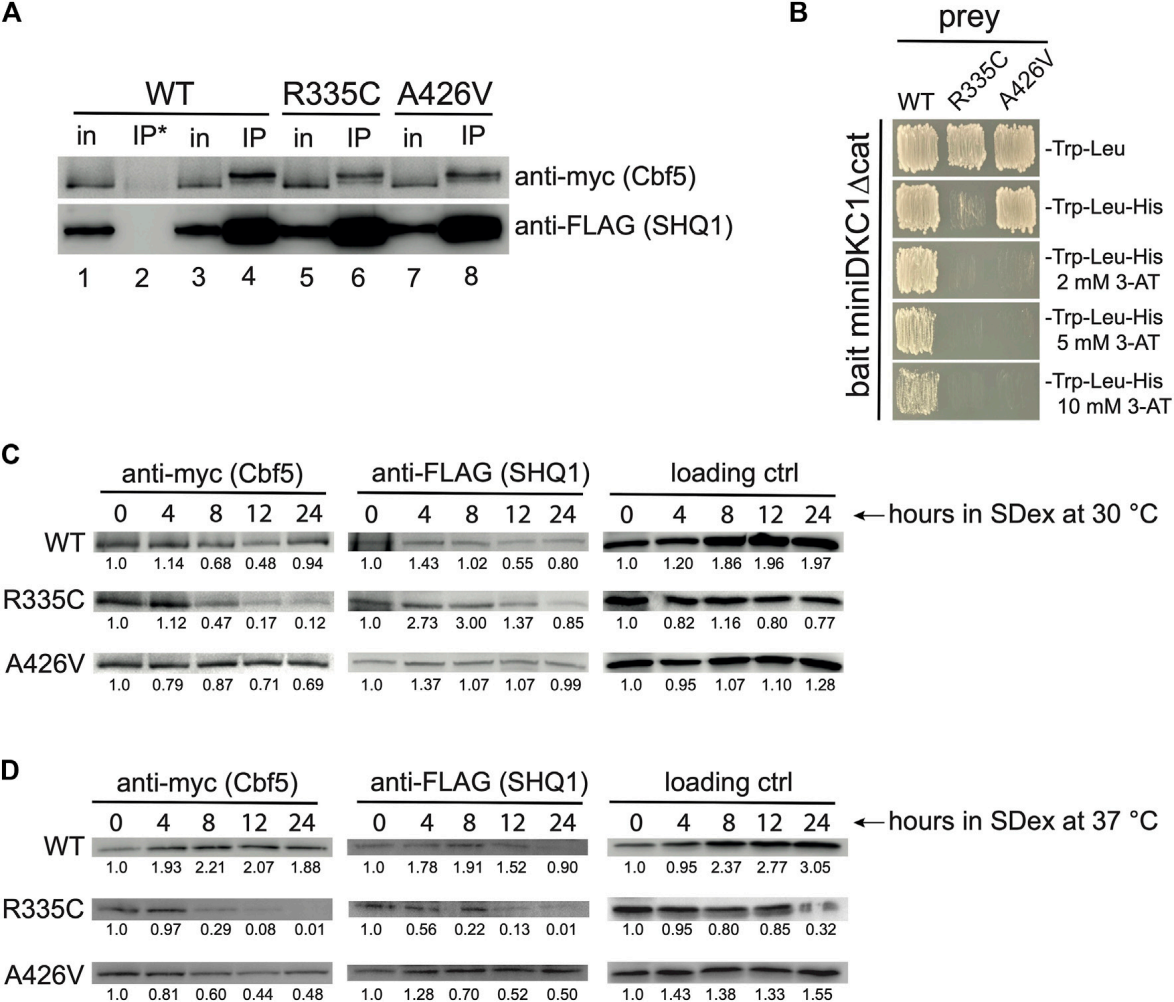
Weakened SHQ1-dyskerin interaction. **(A)** Western blots of IPs done with anti-FLAG beads (IP) or uncoated beads (IP*) using protein extracts of strain YSO12 expressing plasmid-borne human SHQ1 (WT) or its variants (R335C and A426V). For comparison, 5% of the input protein extract (in) was loaded on the gel. The slower migration of myc-tagged Cbf5 in IP lanes has been observed previously with human dyskerin (Wang and Meier, 2004; Trahan et al., 2010); this could be a migration artifact, or it could result from post-translational modifications on Cbf5/dyskerin, such as sumoylation or phosphorylation (Cherry et al., 2012; Garus and Autexier, 2021; Oughtred et al., 2021). **(B)** Y2H assays using miniDKC1∆cat as bait and WT hSHQ1 or its variants as prey. Interaction between bait and prey was monitored by growth on medium lacking histidine; addition of 3-AT allowed visualization of the strongest two-hybrid interactions. **(C)** Western blots made with extracts prepared from strains cultivated in SDex-Ura at 30°C for different duration. Blots were incubated with anti-myc mAb to detect Cbf5 and anti-FLAG mAb to detect hSHQ1 or its variants (indicated on the left). The 50-kDa protein that cross-reacts with 12CA5 anti-HA mAb was used as loading control (see Figure 1). For each panel bands were quantified with ImageJ, and ratios calculated relative to time 0 h in SDex. **(D)** Western blots were made as in **(C)**, except that extracts were prepared from strains cultivated at 37°C. Bands were quantified as in **(C)**.

Pulldown assays with bacterially expressed recombinant proteins revealed that mutations R335C and A426V in SHQ1 disrupt the interaction with dyskerin, mutation R335C having a more severe effect than A426V (Bizarro and Meier, 2017). To determine if such defects exist *in vivo*, we carried out immunoprecipitation experiments (IPs) with extracts prepared from yeast cells expressing WT hSHQ1 or its variants. Note that extracts were prepared from cells shifted to SDex-Ura for only 2 hours, a time point where normal growth was maintained (Figure 1C). IPs were done with anti-FLAG beads and analyzed by Western blotting (Figure 2A). These experiments suggested that the two variants interacted less well with yeast pseudouridine synthase Cbf5 compared to WT hSHQ1. Considering the efficiency of anti-FLAG IPs (see anti-FLAG panel in Figure 2A), it appeared that variant R335C co-immunoprecipitated less efficiently than variant A426V. Results of IPs were corroborated by yeast two-hybrid (Y2H) assays using a truncated version of dyskerin as bait (miniDKC1∆cat; see Supplementary Figure S2 for details) and WT hSHQ1 or its variants as prey (Figure 2B). The stringency of Y2H assays was enhanced by addition of increasing amounts of 3-AT (Toby and Golemis, 2001; Soltanieh et al., 2014). Y2H assays confirmed the previous observation that mutation R335C severely perturbed binding to dyskerin, whereas mutation A426V was less detrimental (Bizarro and Meier, 2017).

We also monitored the steady-state levels of Cbf5 by Western blotting with extracts from cells cultivated in SDex-Ura at 30°C for up to 24 h. These experiments clearly indicated that Cbf5 did not properly accumulate in cells expressing variant R335C; indeed, the signal for myc-tagged Cbf5 markedly decreased 8 h after the shift to SDex-Ura, getting even weaker at later time points (Figure 2C; intensity of bands was quantified relative to time 0 h). This phenomenon was exacerbated when cells expressing variant R335C were cultivated at 37°C (Figure 2D). Levels of Cbf5 did not markedly decrease in mutant A426V cultivated at 30°C (Figure 2C), however, they diminished more significantly when cultivated at 37°C (Figure 2D). The data are consistent with an under accumulation of Cbf5 in strains expressing SHQ1 variants.

### 3.3 Mutations in hSHQ1 impair accumulation of H/ACA snoRNAs

**FIGURE 3:**
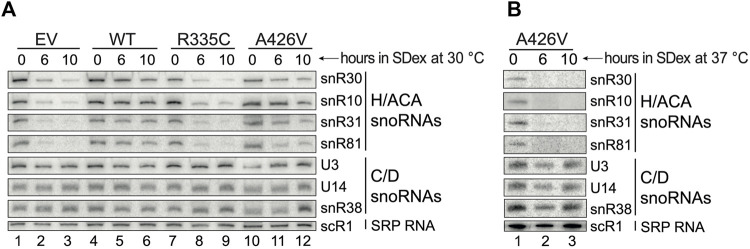
Accumulation of H/ACA snoRNAs is compromised under restrictive conditions. **(A)** Northern hybridization analyses of small RNAs isolated from cells cultivated in SDex-Ura at 30°C and expressing WT hSHQ1 (lanes 4–6) or variants R335C (lanes 7–9) and A426V (lanes 10–12). Complementation with the empty vector (EV) served as negative control (lanes 1–3). Membranes were probed for snoRNAs of the H/ACA class and of the C/D class, and scR1 (cytoplasmic SRP), as indicated on the right. **(B)** Northern hybridizations were done as in **(A)** except that small RNAs were isolated from cells cultivated at 37°C.

Given that mutations in hSHQ1 decreased the levels of yeast Cbf5 (Figures 2C, D), we examined whether this could also impact the stability of H/ACA snoRNAs. To this end, small RNAs were extracted from the different yeast strains cultivated in SDex-Ura for 6 and 10 h, and they were analyzed by northern hybridization (Figure 3); these depletion times were chosen based on the growth curves presented in Figure 1C. In a first series of experiments, yeast cells were cultivated at the optimal temperature of 30°C (Figure 3A). As expected, cells transformed with the empty vector (EV) showed rapid loss of H/ACA snoRNAs when shifted from SGal-Ura to SDex-Ura medium (compare lane 1 with lanes 2 and 3), but there was no change in abundance of C/D snoRNAs or cytoplasmic scR1, the RNA component of the signal recognition particle (SRP). Cells expressing WT hSHQ1 maintained normal levels of all small RNAs tested (lanes 4–6). In marked contrast, H/ACA snoRNA levels were heavily reduced in cells expressing the R335C variant, just as seen with cells transformed with the EV (compare lanes 7-9 with lanes 1–3). Accumulation of H/ACA snoRNAs was only slightly reduced in cells complemented with variant A426V when incubated at 30°C; when incubated at 37°C, however, these cells could no longer accumulate H/ACA snoRNAs but the C/D snoRNAs were not affected (Figure 3B). These results highlight the very specific defects of hSHQ1 variants on accumulation of H/ACA snoRNAs.

### 3.4 Perturbation of pre-rRNA processing and ribosome biogenesis

**FIGURE 4:**
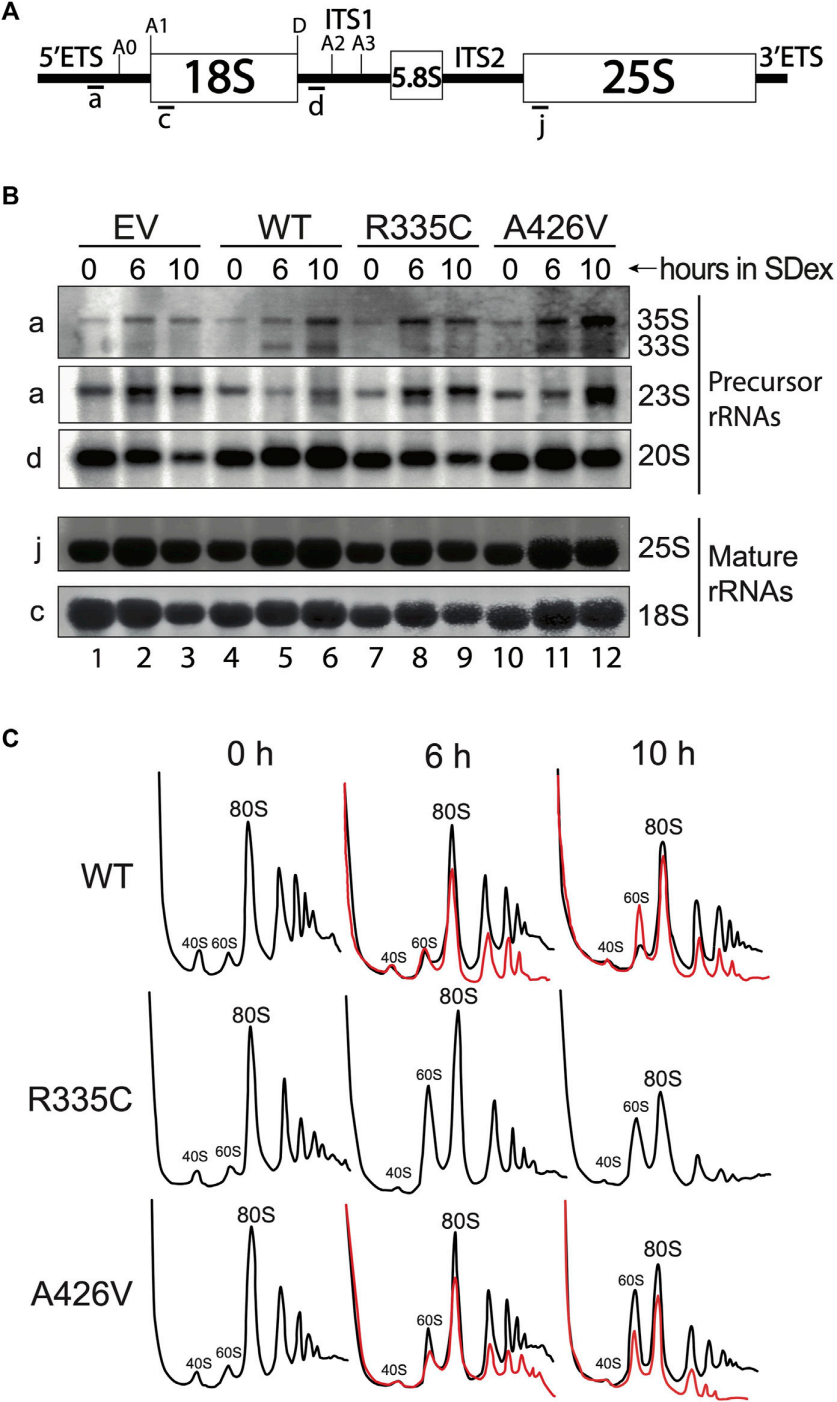
Defects in pre-rRNA processing and ribosome production. **(A)** Schematic of the 35S pre-rRNA. The position of hybridization of the various probes is indicated below the 35S pre-rRNA. For simplicity, only the relevant cleavage sites are indicated. **(B)** Northern blots made with total RNA isolated from yeast strains cultivated in SDex at 30°C. Strain YSO12 was complemented with the empty vector (EV; lanes 1–3), WT hSHQ1 (lanes 4–6), variant R335C (lanes 7–9) or variant A426V (10–12). Time of depletion in SDex is indicated above each lane. Oligonucleotide probes used for blotting are shown on the left, and rRNA species are indicated on the right. **(C)** Sucrose density gradient analyses of ribosomal particles. Cell extracts were prepared from strain YSO12 complemented with WT hSHQ1 (top panel), variant R335C (middle panel) or variant A426V (bottom panel). The profiles were obtained by continuous monitoring of the absorbance at 254 nm. Strains were cultivated in SDex at 30°C (black lines) or 37°C (red lines). Peaks corresponding to 40S and 60S subunits, 80S ribosomes and polysomes are indicated.

The strong decrease in accumulation of H/ACA snoRNAs could result in pre-rRNA processing defects and reduced production of ribosomes. These possibilities were investigated by Northern blotting of rRNAs and sucrose density gradients (Figure 4). Total RNA was extracted from yeast strains incubated at 30°C in SDex-Ura for up to 10 h. Northern hybridization was carried out with ^32^P-labelled oligonucleotide probes that hybridize to specific regions of the pre-rRNA (Figure 4A). Complementation of strain YSO12 with the empty vector resulted in pre-rRNA processing defects seen upon inhibition of the early cleavage reactions, i.e., the cleavages at sites A0 and A1 in the 5′ETS, and site A2 in ITS1. Cleavage inhibition at these three sites led to accumulation of the 23S precursor, which was produced by endonucleolytic cleavage at site A3 in ITS1 (the 23S extends from the 5′-end to site A3). Moreover, levels of the 20S precursor were reduced in those cells (compare lanes 1 and 3 in Figure 4B). The 20S pre-rRNA extends from site A1 to site A2; this precursor is normally exported to the cytoplasm for final maturation at site D, which produces mature 18S rRNA (Supplementary Figure S1). Concomitant with reduced 20S levels, there was a decrease of 18S rRNA levels. All those processing defects were not observed when strain YSO12 was complemented with WT hSHQ1 (lanes 4 to 6 in Figure 4B); pre-rRNA processing in those cells followed the normal pathway (see Supplementary Figure S1). Complementation with variant R335C recapitulated the processing defects seen with the EV (Figure 4B: compare lanes 7 to 9 with lanes 1–3). When strain YSO12 was complemented with variant A426V, pre-rRNA processing appeared normal although somewhat delayed: in comparison with the WT, variant A426V accumulated 35S and 23S precursors, and we detected less 20S pre-rRNA (compare lanes 10–12 with WT lanes 4–6). In line the temperature-sensitivity observed with strain A426V (Figures 1, 3), pre-rRNA processing was compromised when cells were cultivated at 37°C (Supplementary Figure S3).

Analyses on sucrose density gradients confirmed that pre-rRNA processing defects lead to impaired ribosome production (Figure 4C). With WT hSHQ1 the profiles remained normal after the shift from SGal (0 h) to SDex medium. In contrast, variant R335C presented an important change only 6 h after the shift to SDex: the enlarged peak of 60S subunits is indicative of imbalanced 40S:60S stoichiometry (shortage of 40S subunits). Most importantly, peaks for 80S ribosomes and polysomes were greatly reduced 10 h after the shift to SDex (Figure 4C, middle row). Complementation with variant A426V at 30°C led to a mild alteration of sedimentation profiles (Figure 4C, bottom row, black curves). However, cultivating this strain at 37°C largely altered the sedimentation profiles, including a strong loss of polysomes (Figure 4C, bottom row, red curves). Polysome profiles were not altered as significantly with the WT strain over time (compare the red line profiles of WT and A426V in Figure 4C), even though the WT strain did not grow optimally when incubated at 37°C (Figure 1E; Sleiman et al., 2022). Therefore, the polysome defects observed with strain A426V likely result from perturbed function of this SHQ1 variant.

## 4 Discussion

Several studies in yeast and mammalian cells have illustrated the important role of SHQ1 in chaperoning the pseudouridine synthase Cbf5/dyskerin, the catalytic component of H/ACA snoRNPs responsible for site-directed pseudouridylation of ribosomal RNAs (reviewed in Yu and Meier, 2014). Here we investigated two compound heterozygous variants in human *SHQ1* that were identified in a child presenting severe neurological disorder (Bizarro and Meier, 2017). In this original report, variants p. R335C and p. A426V showed reduced *in vitro* binding affinity for dyskerin. To elucidate the molecular events altered by mutations in SHQ1 *in vivo*, we used a haploid yeast model system that allows conditional expression of the endogenous yeast Shq1 protein while constitutively expressing plasmid-borne human SHQ1 (WT or variants). Variants R335C and A426V were individually tested in our yeast model.

In accordance with the *in vitro* binding assays showing weaker interaction of variant p. R335C to dyskerin compared to variant p. A426V (Bizarro and Meier, 2017), we found that mutation R335C is more deleterious than mutation A426V *in vivo*. The yeast strain expressing R335C was unviable when cells were depleted of endogenous yeast Shq1, and Western blotting revealed that levels of Cbf5 were reduced under these conditions. This could explain why H/ACA snoRNAs, but not C/D snoRNAs, did not accumulate in this strain. Since snR30 is the only H/ACA snoRNA required for the cleavage reactions at sites A0-A2 on the pre-rRNA, the early pre-rRNA processing defects observed in the R335C strain are attributable to the sole absence of snR30. Nonetheless, the absence of all the other H/ACA snoRNAs, which guide *Ψ f*ormation in critical regions of rRNAs, could also impact ribosomal subunit formation because unmodified rRNAs are unstable (Charette and Gray, 2000; Ofengand, 2002; King et al., 2003; Liang et al., 2007; Sloan et al., 2017), and we observed less ribosomes and polysomes at longer depletion times (Figure 4C). Expression of variant p. A426V in yeast led to intermediate growth defects when cells were cultivated at low temperatures, but the mutation was lethal at 37°C (Figure 1). In line with this temperature-sensitive phenotype, H/ACA snoRNAs did not accumulate at 37°C, which caused severe pre-rRNA processing defects and important loss of ribosomes and polysomes. In contrast, mild growth defects were observed at 30°C and accumulation of H/ACA snoRNAs appeared marginally altered. Still, pre-rRNA processing was less efficient in those cells and the production of ribosomes was slightly reduced (Figure 4).

The striking phenotypic differences caused by expression of hSHQ1 variants R335C or A426V likely result from the location and function of the mutated residues. To evaluate the damaging effects of these amino acids changes, we carried out analyses with the predictive algorithms PolyPhen-2 (Adzhubei et al., 2010) and SIFT (Sim et al., 2012). PolyPhen-2 predicted that mutations R335C and A426V likely have profound effects (HumVar score of 0.999 and 0.996, respectively). Analyses with SIFT indicated that only an arginine residue would be tolerated at position 335, supporting the very strong phenotype observed with variant R335C. In contrast, A426 could apparently be replaced by other amino acids, including a valine. This latter result is consistent with the milder phenotype observed with mutant A426V. Arginine 335 is very highly conserved (Li et al., 2011), underscoring its functional importance. Its yeast counterpart (R383) is in the loop of a helix-turn-helix; this loop is positioned at the interface with Cbf5, where R383 interacts with residues Y281 and D344, both of which are conserved in Cbf5 orthologs (Li et al., 2011; Walbott et al., 2011). Changing R335 to a cysteine likely precludes these interactions and the tight binding of hSHQ1 to Cbf5. This is supported by *in vitro* binding assays (Bizarro and Meier, 2017), and the reduced co-IP of Cbf5 with human SHQ1 bearing mutation R335C (Figure 2A). Regarding mutation A426V in human SHQ1, making comparisons with its yeast ortholog is not straightforward: published sequence alignments indicate that A426 could be equivalent to N475 (Walbott et al., 2011) or to T485 (Li et al., 2011), both of which are part of a β hairpin. However, the primary sequence of this region is poorly conserved between yeast Shq1 and mammalian orthologs, and it is unclear whether the β hairpin exists in human SHQ1. To address this issue, we carried out structure predictions of human SHQ1 with Phyre2 (Kelley et al., 2015) and I-TASSER (Yang and Zhang, 2015), and we searched the AlphaFold Protein Structure Database (Varadi et al., 2022). Structures obtained with Phyre and AlphaFold suggest that A426 is in a long *a* helix, whereas I-TASSER places A426 in a U-shaped loop that precedes an *a* helix. This latter configuration is reminiscent of the β hairpin and neighboring C-terminal helix of yeast Shq1, which form a V-shaped groove that binds the C-terminal helix of Cbf5 (Li et al., 2011; Walbott et al., 2011). Although this C-terminal *a* helix exists in human dyskerin (Wan et al., 2021; Liu et al., 2022), structural studies will be required to determine if it interacts with a similar V-shaped groove in human SHQ1. If this were the case, the phenotype conferred by mutation A426V could result from perturbation of the U-shaped loop (or β hairpin) at high temperatures. Overall, the primary cause of growth defects with SHQ1 variants roots in the weakened SHQ1-Cbf5 interaction.

The first SHQ1 variants were identified in a patient that suffered from intrauterine growth retardation and a neurological disorder comprising cerebellar degeneration, which were evocative of the Hoyeraal-Hreidarsson syndrome, a severe form of dyskeratosis congenita (DC); however, it had not been possible to verify if the patient had short telomeres, a hallmark of DC (Bizarro and Meier, 2017). More recent reports have linked SHQ1 mutations to global developmental delays, seizures and dystonia (Sleiman et al., 2022; AlHargan et al., 2023; Chi et al., 2023; Indelicato et al., 2023). Thus, mutations in SHQ1 have a strong impact on neurodevelopment, and further investigations in higher model organisms should highlight the full spectrum of disorders during development. Given that *SHQ1* variants altering formation of H/ACA snoRNPs and ribosomes are linked to dystonia, this syndrome should be added to the list of ribosomopathies.

## Data Availability

The original contributions presented in the study are included in the article/Supplementary Material, further inquiries can be directed to the corresponding author.

## References

[B1] Adzhubei I. A. Schmidt S. Peshkin L. Ramensky V. E. Gerasimova A. Bork P. (2010). A method and server for predicting damaging missense mutations. Nat. Methods. 7, 248–249. 10.1038/nmeth0410-248 20354512 PMC2855889

[B2] AlHargan A. AlMuhaizea M. A. Almass R. Alwadei A. H. Daghestani M. Arold S. T. (2023). SHQ1-associated neurodevelopmental disorder: report of the first homozygous variant in unrelated patients and review of the literature. Hum. Genome Var. 10, 7. 10.1038/s41439-023-00234-z 36810590 PMC9944922

[B3] Armistead J. Triggs-Raine B. (2014). Diverse diseases from a ubiquitous process: the ribosomopathy paradox. FEBS Lett. 588, 1491–1500. 10.1016/j.febslet.2014.03.024 24657617

[B4] Aubert M. O'Donohue M. F. Lebaron S. Gleizes P. E. (2018). Pre-ribosomal RNA processing in human cells: from Mechanisms to congenital diseases. Biomolecules 8, 123. 10.3390/biom8040123 30356013 PMC6315592

[B5] Bassler J. Hurt E. (2019). Eukaryotic ribosome assembly. Annu. Rev. Biochem. 88, 281–306. 10.1146/annurev-biochem-013118-110817 30566372

[B6] Bizarro J. Meier U. T. (2017). Inherited SHQ1 mutations impair interaction with NAP57/dyskerin, a major target in dyskeratosis congenita. Mol. Genet. Genomic Med. 5, 805–808. 10.1002/mgg3.314 29178645 PMC5702568

[B7] Charette M. Gray M. W. (2000). Pseudouridine in RNA: what, where, how, and why. IUBMB Life 49, 341–351. 10.1080/152165400410182 10902565

[B8] Cherry J. M. Hong E. L. Amundsen C. Balakrishnan R. Binkley G. Chan E. T. (2012). Saccharomyces Genome Database: the genomics resource of budding yeast. Nucleic Acids Res. 40, D700–D705. 10.1093/nar/gkr1029 22110037 PMC3245034

[B9] Chi C. S. Tsai C. R. Lee H. F. (2023). Biallelic SHQ1 variants in early infantile hypotonia and paroxysmal dystonia as the leading manifestation. Hum. Genet. 142, 1029–1041. 10.1007/s00439-023-02533-5 36847845

[B10] Ciganda M. Williams N. (2011). Eukaryotic 5S rRNA biogenesis. Wiley Interdiscip. Rev. RNA. 2, 523–533. 10.1002/wrna.74 21957041 PMC3278907

[B11] Decatur W. A. Fournier M. J. (2002). rRNA modifications and ribosome function. Trends biochem. Sci. 27, 344–351. 10.1016/s0968-0004(02)02109-6 12114023

[B12] Farley-Barnes K. I. Ogawa L. M. Baserga S. J. (2019). Ribosomopathies: old concepts, new controversies. Trends Genet. 35, 754–767. 10.1016/j.tig.2019.07.004 31376929 PMC6852887

[B13] Garí E. Piedrafita L. Aldea M. Herrero E. (1997). A set of vectors with a tetracycline-regulatable promoter system for modulated gene expression in *Saccharomyces cerevisiae* . Yeast 13, 837–848. 10.1002/(SICI)1097-0061(199707)13:9<837::AID-YEA145>3.0.CO;2-T 9234672

[B14] Garus A. Autexier C. (2021). Dyskerin: an essential pseudouridine synthase with multifaceted roles in ribosome biogenesis, splicing, and telomere maintenance. RNA 27, 1441–1458. 10.1261/rna.078953.121 34556550 PMC8594475

[B15] Gietz R. D. Woods R. A. (2002). Transformation of yeast by lithium acetate/single-stranded carrier DNA/polyethylene glycol method. Methods Enzymol. 350, 87–96. 10.1016/s0076-6879(02)50957-5 12073338

[B16] Higuchi R. Krummel B. Saiki R. K. (1988). A general method of *in vitro* preparation and specific mutagenesis of DNA fragments: study of protein and DNA interactions. Nucleic Acids Res. 16, 7351–7367. 10.1093/nar/16.15.7351 3045756 PMC338413

[B17] Indelicato E. Boesch S. Baumgartner M. Plecko B. Winkelmann J. Zech M. (2023). Confirmation of a causal role for SHQ1 variants in early infantile-onset recessive dystonia. Mov. Disord. 38, 355–357. 10.1002/mds.29281 36416405

[B18] Kelley L. A. Mezulis S. Yates C. M. Wass M. N. Sternberg M. J. (2015). The Phyre2 web portal for protein modeling, prediction and analysis. Nat. Protoc. 10, 845–858. 10.1038/nprot.2015.053 25950237 PMC5298202

[B19] King T. H. Liu B. McCully R. R. Fournier M. J. (2003). Ribosome structure and activity are altered in cells lacking snoRNPs that form pseudouridines in the peptidyl transferase center. Mol. Cell. 11, 425–435. 10.1016/s1097-2765(03)00040-6 12620230

[B20] Klinge S. Woolford J. L. Jr. (2019). Ribosome assembly coming into focus. Nat. Rev. Mol. Cell Biol. 20, 116–131. 10.1038/s41580-018-0078-y 30467428 PMC7725133

[B21] Kressler D. Hurt E. Bassler J. (2010). Driving ribosome assembly. Biochim. Biophys. Acta. 1803, 673–683. 10.1016/j.bbamcr.2009.10.009 19879902

[B22] Lafontaine D. L. Tollervey D. (2001). The function and synthesis of ribosomes. Nat. Rev. Mol. Cell Biol. 2, 514–520. 10.1038/35080045 11433365

[B23] Lemay V. Hossain A. Osheim Y. N. Beyer A. L. Dragon F. (2011). Identification of novel proteins associated with yeast snR30 small nucleolar RNA. Nucleic Acids Res. 39, 9659–9670. 10.1093/nar/gkr659 21893585 PMC3239182

[B24] Li S. Duan J. Li D. Ma S. Ye K. (2011). Structure of the Shq1-Cbf5-Nop10-Gar1 complex and implications for H/ACA RNP biogenesis and dyskeratosis congenita. EMBO J. 30, 5010–5020. 10.1038/emboj.2011.427 22117216 PMC3242979

[B25] Liang X. H. Liu Q. Fournier M. J. (2007). rRNA modifications in an intersubunit bridge of the ribosome strongly affect both ribosome biogenesis and activity. Mol. Cell. 28, 965–977. 10.1016/j.molcel.2007.10.012 18158895

[B26] Liu B. He Y. Wang Y. Song H. Zhou Z. H. Feigon J. (2022). Structure of active human telomerase with telomere shelterin protein TPP1. Nature 604, 578–583. 10.1038/s41586-022-04582-8 35418675 PMC9912816

[B27] Massenet S. Bertrand E. Verheggen C. (2017). Assembly and trafficking of box C/D and H/ACA snoRNPs. RNA Biol. 14, 680–692. 10.1080/15476286.2016.1243646 27715451 PMC5519232

[B28] Maxwell E. S. Fournier M. J. (1995). The small nucleolar RNAs. Annu. Rev. Biochem. 64, 897–934. 10.1146/annurev.bi.64.070195.004341 7574504

[B29] Narla A. Ebert B. L. (2010). Ribosomopathies: human disorders of ribosome dysfunction. Blood 115, 3196–3205. 10.1182/blood-2009-10-178129 20194897 PMC2858486

[B30] Ofengand J. (2002). Ribosomal RNA pseudouridines and pseudouridine synthases. FEBS Lett. 514, 17–25. 10.1016/s0014-5793(02)02305-0 11904174

[B31] Oughtred R. Rust J. Chang C. Breitkreutz B. J. Stark C. Willems A. (2021). The BioGRID database: A comprehensive biomedical resource of curated protein, genetic, and chemical interactions. Protein Sci. 30, 187–200. 10.1002/pro.3978 33070389 PMC7737760

[B32] Panov K. I. Hannan K. Hannan R. D. Hein N. (2021). The ribosomal gene loci-the power behind the throne. Genes (Basel) 12, 763. 10.3390/genes12050763 34069807 PMC8157237

[B33] Schlotter F. Merouani S. Flayac J. Kogey V. Issa A. Dodre M. (2023). Proteomic analyses reveal new features of the box H/ACA RNP biogenesis. Nucleic Acids Res. 51, 3357–3374. 10.1093/nar/gkad129 36869663 PMC10123114

[B34] Schneider C. A. Rasband W. S. Eliceiri K. W. (2012). NIH image to ImageJ: 25 years of image analysis. Nat. Methods. 9, 671–675. 10.1038/nmeth.2089 22930834 PMC5554542

[B35] Sharma S. Lafontaine D. L. J. (2015). 'View from A bridge': A new perspective on eukaryotic rRNA base modification. Trends biochem. Sci. 40, 560–575. 10.1016/j.tibs.2015.07.008 26410597

[B36] Sim N. L. Kumar P. Hu J. Henikoff S. Schneider G. Ng P. C. (2012). SIFT web server: predicting effects of amino acid substitutions on proteins. Nucleic Acids Res. 40, W452–W457. 10.1093/nar/gks539 22689647 PMC3394338

[B37] Sleiman S. Marshall A. E. Dong X. Mhanni A. Alidou-D'Anjou I. Frosk P. (2022). Compound heterozygous variants in SHQ1 are associated with a spectrum of neurological features, including early-onset dystonia. Hum. Mol. Genet. 31, 614–624. 10.1093/hmg/ddab247 34542157

[B38] Sloan K. E. Warda A. S. Sharma S. Entian K. D. Lafontaine D. L. J. Bohnsack M. T. (2017). Tuning the ribosome: the influence of rRNA modification on eukaryotic ribosome biogenesis and function. RNA Biol. 14, 1138–1152. 10.1080/15476286.2016.1259781 27911188 PMC5699541

[B39] Soltanieh S. Lapensée M. Dragon F. (2014). Nucleolar proteins Bfr2 and Enp2 interact with DEAD-box RNA helicase Dbp4 in two different complexes. Nucleic Acids Res. 42, 3194–3206. 10.1093/nar/gkt1293 24357410 PMC3950691

[B40] Soltanieh S. Osheim Y. N. Spasov K. Trahan C. Beyer A. L. Dragon F. (2015). DEAD-box RNA helicase Dbp4 is required for small-subunit processome formation and function. Mol. Cell. Biol. 35, 816–830. 10.1128/MCB.01348-14 25535329 PMC4323488

[B41] Toby G. G. Golemis E. A. (2001). Using the yeast interaction trap and other two-hybrid-based approaches to study protein-protein interactions. Methods 24, 201–217. 10.1006/meth.2001.1182 11403570

[B42] Trahan C. Dragon F. (2009). Dyskeratosis congenita mutations in the H/ACA domain of human telomerase RNA affect its assembly into a pre-RNP. RNA 15, 235–243. 10.1261/rna.1354009 19095616 PMC2648702

[B43] Trahan C. Martel C. Dragon F. (2010). Effects of dyskeratosis congenita mutations in dyskerin, NHP2 and NOP10 on assembly of H/ACA pre-RNPs. Hum. Mol. Genet. 19, 825–836. 10.1093/hmg/ddp551 20008900

[B44] Varadi M. Anyango S. Deshpande M. Nair S. Natassia C. Yordanova G. (2022). AlphaFold protein structure database: massively expanding the structural coverage of protein-sequence space with high-accuracy models. Nucleic Acids Res. 50, D439–D444. 10.1093/nar/gkab1061 34791371 PMC8728224

[B45] Venturi G. Montanaro L. (2020). How altered ribosome production can cause or contribute to human disease: the spectrum of ribosomopathies. Cells 9, 2300. 10.3390/cells9102300 33076379 PMC7602531

[B46] Walbott H. Machado-Pinilla R. Liger D. Blaud M. Rety S. Grozdanov P. N. (2011). The H/ACA RNP assembly factor SHQ1 functions as an RNA mimic. Genes Dev. 25, 2398–2408. 10.1101/gad.176834.111 22085966 PMC3222905

[B47] Wan F. Ding Y. Zhang Y. Wu Z. Li S. Yang L. (2021). Zipper head mechanism of telomere synthesis by human telomerase. Cell Res. 31, 1275–1290. 10.1038/s41422-021-00586-7 34782750 PMC8648750

[B48] Wang C. Meier U. T. (2004). Architecture and assembly of mammalian H/ACA small nucleolar and telomerase ribonucleoproteins. EMBO J. 23, 1857–1867. 10.1038/sj.emboj.7600181 15044956 PMC394235

[B49] Watkins N. J. Bohnsack M. T. (2012). The box C/D and H/ACA snoRNPs: key players in the modification, processing and the dynamic folding of ribosomal RNA. Wiley Interdiscip. Rev. RNA. 3, 397–414. 10.1002/wrna.117 22065625

[B50] Woolford J. L. Jr. Baserga S. J. (2013). Ribosome biogenesis in the yeast *Saccharomyces cerevisiae* . Genetics 195, 643–681. 10.1534/genetics.113.153197 24190922 PMC3813855

[B51] Yang J. Zhang Y. (2015). I-TASSER server: new development for protein structure and function predictions. Nucleic Acids Res. 43, W174–W181. 10.1093/nar/gkv342 25883148 PMC4489253

[B52] Yelick P. C. Trainor P. A. (2015). Ribosomopathies: global process, tissue specific defects. Rare Dis. 3, e1025185. 10.1080/21675511.2015.1025185 26442198 PMC4590025

[B53] Yu Y. T. Meier U. T. (2014). RNA-guided isomerization of uridine to pseudouridine--pseudouridylation. RNA Biol. 11, 1483–1494. 10.4161/15476286.2014.972855 25590339 PMC4615163

