# Supplementary material for: Human SHQ1 variants R335C and A426V lead to severe ribosome biogenesis defects when expressed in yeast
Source: Front Genet. 2023 Sep 25;14:1240416. doi: 10.3389/fgene.2023.1240416 (PMC10560722; doi:10.3389/fgene.2023.1240416)
Supplement: Supplementary file 1 [file DataSheet1.PDF]

Supplementary Material

Table S1. Yeast strains

Strain	Genotype	Reference/source
YPH499	*Mat*a, *ura3-52*, *lys2-801*, *ade2-101*, *trp1-Δ63*, *his3-Δ200*, *leu2-Δ1*	Sikorski and Hieter, 1989
AH109	*Mat*a, *trp1-901*, *leu2-3*, *112*, *ura3-52*, *his3-200*, *gal4Δ*, *gal80Δ*, *LYS2::GAL1*$_{UAS}$*-GAL1*$_{TATA}$*-HIS3*, *GAL2*$_{UAS}$*-GAL2*$_{TATA}$*-ADE2*, *URA3::MEL1*$_{UAS}$*-MEL1*$_{TATA}$*-LacZ*	Clontech
YSO12	Same as YPH499 except *kanMX6*::P$_{GAL1}$*-3HA-SHQ1*, *CBF5::CBF5-9MYC-TRP1*	Sleiman et al., 2022
YSO13	YSO12 carrying empty plasmid pCM-FLAG	Sleiman et al., 2022
YSO14	YSO12 carrying plasmid pCM-hSHQ1-FLAG	Sleiman et al., 2022
YAD1	YSO12 carrying plasmid pCM-R335C-FLAG	This study
YAD2	YSO12 carrying plasmid pCM-A426V-FLAG	This study
YAD3	AH109 carrying plasmids pGBK-miniDKC1Δcat and pGAD-hSHQ1	This study
YAD4	AH109 carrying plasmids pGBK-miniDKC1Δcat and pGAD-R335C	This study
YAD5	AH109 carrying plasmids pGBK-miniDKC1Δcat and pGAD-A426V	This study

Table S2. Oligonucleotides used for northern hybridization.

Primer	Description	Sequence (5'-3')
FD4	anti-snR10 (H/ACA)	CAATCCTTGCAACGGTCCTCATCC
FD6	anti-snR30 (H/ACA)	GGAATATACTGCGGTAGGACGAAC
FD9	anti-scR1 (SRP)	CCCACCAGAAAGCCATTACAGCC
FD10	anti-yU3 (C/D)	CATAGGATGGGTCAAGATCATCGCGCC
FD11	anti-yU14 (C/D)	CGATGGGTTCGTAAGCGTACTCCTACCGTGG
FD533	anti-snR38 (C/D)	GAGGTTACCTATTATTACCC
FD537	anti-snR31 (H/ACA)	CAACGCCCATCAAACATCCG
FD1500	anti-snR81 (H/ACA)	ACTTGATGTGGGCTGCCTCG
FD529	Probe a (5'-A0)	CGCTGCTCACCAATGG
FD106	Probe c (anti-18S)	CATGGCTTAATCTTTGAGAC
FD565	Probe d (D-A2)	GCTCTCATGCTCTTGCC
FD107	Probe j (anti-25S)	CTCCGCTTATTGATATGC

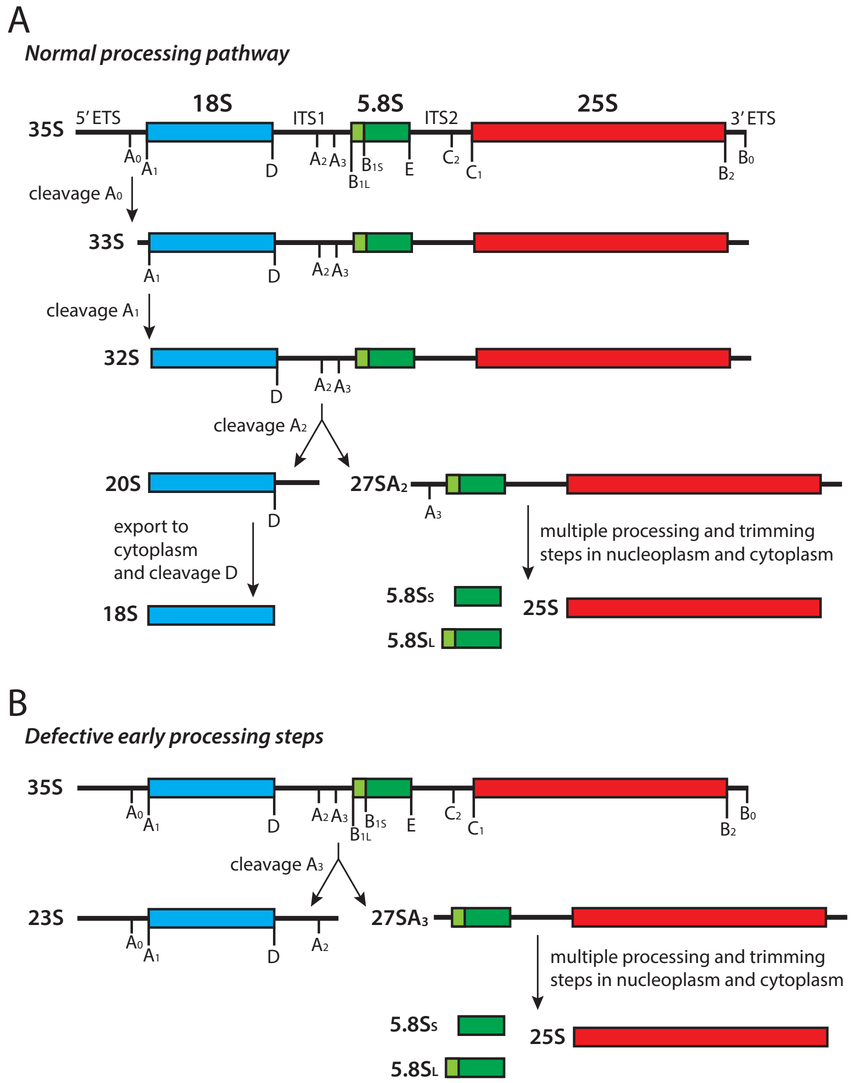

Figure S1. Pre-rRNA processing pathway in *Saccharomyces cerevisiae*.

(A) Simplified scheme of the normal processing pathway. The 35S precursor encodes three rRNAs: 18S (blue), 5.8S (green), and 25S (red). This precursor is subjected to early processing steps (cleavages at sites A0, A1 and A2) that liberate two precursors, one of 20S for the small ribosomal subunit (SSU) and one of 27S for the large ribosomal subunit (LSU). The 20S precursor is exported from the nucleolus to the cytoplasm, where cleavage at site D generates mature 18S rRNA of the SSU. The 27SA2 precursor is subjected to multiple processing and trimming steps occurring in different cellular compartments. These maturations steps will generate the 25S rRNA and the 5.8S rRNA, which can be found in the short (5.8SS) or long (5.8SL) form in the LSU.

(B) Simplified scheme of the defective processing pathway when the three early processing events are altered. Inhibition of cleavages at sites A0, A1 and A2 produces a precursor of 23S that accumulates in cells, whereas cleavage at site A3 allows normal production of LSU rRNAs.

```
Cbf5      ------------------------------MSKEDFVIKPEAAGASTDTSEWPLLLKNFD      30
DKC1      MADAEVIILPKKHKKKKERKSLPEEDVAEIQHAEEFLIKPESKVAKLDTSQWPLLLKNFD      60
                                             *:*:****:  *. ***:*********

Cbf5      KLLVRSGHYTPIPAGSSPLKRDLKSYISSGVINLDKPSNPSSHEVVAWIKRILRCEKTGH      90
DKC1      KLNVRTTHYTPLACGSNPLKREIGDYIRTGFINLDKPSNPSSHEVVAWIRRILRVEKTGH      120
          ** **: ****: .**.****:: .** :*.******************:**** *****

Cbf5      SGTLDPKVTGCLIVCIDRATRLVKSQQGAGKEYVCIVRLHDALKDEKDLGRSLENLTGAL      150
DKC1      SGTLDPKVTGCLIVCIERATRLVKSQQSAGKEYVGIVRLHNAIEGGTQLSRALETLTGAL      180
          ****************:**********.****** *****:*::. .:*.*:**.*****

Cbf5      FQRPPLISAVKRQLRVRTIYESNLIEFDNKRNLGVFWASCEAGTYMRTLCVHLGMLLGVG      210
DKC1      FQRPPLIAAVKRQLRVRTIYESKMIEYDPERRLGIFWVSCEAGTYIRTLCVHLGLLLGVG      240
          *******:**************::**:* :*.**:**.*******:********:*****

Cbf5      GHMQELRRVRSGALSENDNMVTLHDVMDAQWVYDNTRDESYLRSIIQPLETLLVGYKRIV      270
DKC1      GQMQELRRVRSGVMSEKDHMVTMHDVLDAQWLYDNHKDESYLRRVVYPLEKLLTSHKRLV      300
          *:**********.:**:*:***:***:****:*** :****** :: ***.**..:**:*

Cbf5      VKDSAVNAVCYGAKLMIPGLLRYEEGIELYDEIVLITTKGEAIAVAIAQMSTVDLASCDH      330
DKC1      MKDSAVNAICYGAKIMLPGVLRYEDGIEVNQEIVVITTKGEAICMAIALMTTAVISTCDH      360
          :*******:*****:*:**:****:***: :***:********.:*** *:*. :::***

Cbf5      GVVASVKRCIMERDLYPRRWGLGPVAQKKKQMKADGKLDKYGRVNENTPEQWKKEYVPLD      390
DKC1      GIVAKIKRVIMERDTYPRKWGLGPKASQKKLMIKQGLLDKHGKPTDSTPATWKQEYVDYS      420
          *:**.:** ***** ***:***** *.:** *  :* ***:*: .:.**  **:***  .

Cbf5      NAEQSTSSSQETKETEEEPKKAKEDSLIKEVETEKEEVKEDDSKKEKKEKKDKKEKKEKK      450
DKC1      ESAKKEVVAEVVKAPQVVAEAAKTAKRKRESESESDETPPAAPQLIKKEK--------KK      472
          :: :.   :: .*  :   : **  .  :* *:*.:*.     :  ****        **

Cbf5      EKKDKKEKKEKKEKKRKSEDGDSEEKKSKKSKK----------      483
DKC1      SKKDKKA-KAGLESGAEPGDGDSDTTKKKKKKKKAKEVELVSE      514
          .*****  *   *.  :  ****: .*.**.**
```

Figure S2. Sequence alignment of yeast Cbf5 and human dyskerin. Protein sequences of yeast Cbf5 (UniProt P33322) and human dyskerin (DKC1; UniProt O60832) were aligned with Clustal Omega (Madeira et al., 2022. *Nucleic Acids Res.* 50: W276-W279). Segments of DKC1 that were deleted in the construct miniDKC1Δcat used for yeast two-hybrid assays are boxed. Residues deleted in N- and C-terminal regions are in blue. Deleted residues of the catalytic domain are shown in red (replaced by a single glycine).

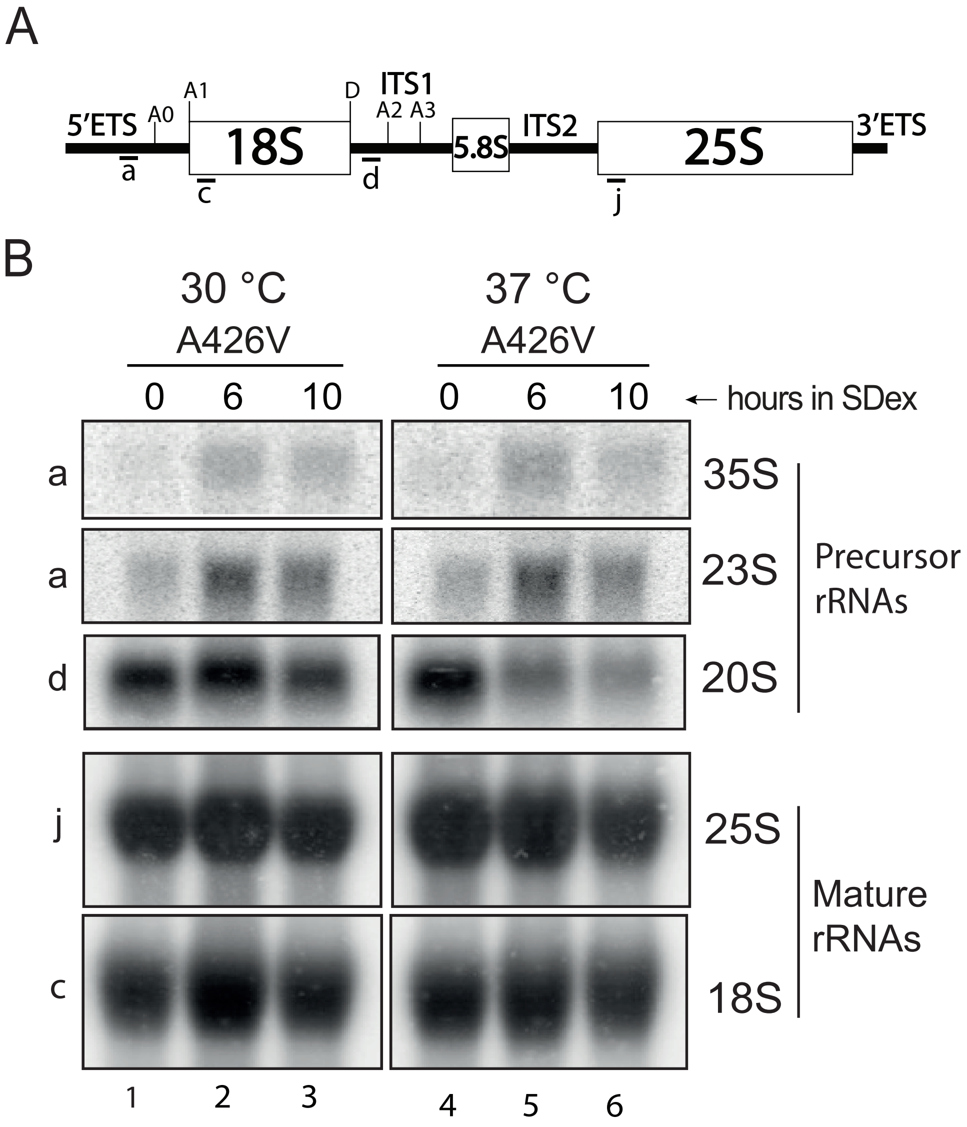

Figure S3. Enhanced processing defects at 37 °C.

(A) Schematic of the 35S pre-rRNA. Position of the various probes hybridizing with 35S pre-rRNA is indicated below the cartoon. Only the relevant cleavage sites are indicated.

(B) Northern blots made with total RNA isolated from yeast strain YSO12 complemented with variant A426V and cultivated in SDex at 30 °C (lanes 1-3) or 37 °C (lanes 4-6). Time of depletion in SDex is indicated above each lane. Oligonucleotide probes used for blotting are shown on the left, and rRNA species are indicated on the right.
